# Deep Learning–Assisted Differentiation of Four Peripheral Neuropathies Using Corneal Confocal Microscopy

**DOI:** 10.1002/acn3.70255

**Published:** 2025-11-22

**Authors:** Chaima Ben Rabah, Ioannis N. Petropoulos, Mark Stettner, Maryam Ferdousi, Uazman Alam, Nathan Efron, Ahmed Serag, Rayaz A. Malik

**Affiliations:** ^1^ AI Innovation Lab Weill Cornell Medicine Doha Qatar; ^2^ Division of Research Weill Cornell Medicine Doha Qatar; ^3^ Department of Neurology and Center for Translational Neuro‐ and Behavioral Sciences (C‐TNBS) University Hospital Essen Essen Germany; ^4^ Faculty of Biology, Medicine and Health University of Manchester Manchester UK; ^5^ Diabetes & Obesity Research, Department of Medicine Aintree University Hospital Liverpool UK; ^6^ Stoke‐on‐Trent, Centre for Biomechanics and Rehabilitation Technologies Staffordshire University UK; ^7^ Institute of Health and Biomedical Innovation Queensland University of Technology Brisbane Australia

**Keywords:** artificial intelligence, corneal confocal microscopy, corneal nerve, disease diagnosis, peripheral neuropathy

## Abstract

**Objective:**

Peripheral neuropathies contribute to patient disability but may be diagnosed late or missed altogether due to late referral, limitation of current diagnostic methods and lack of specialized testing facilities. To address this clinical gap, we developed NeuropathAI, an interpretable deep learning–based multiclass classification system for rapid, automated diagnosis and differentiation of 88 patients with diabetic peripheral neuropathy (DPN), chemotherapy‐induced peripheral neuropathy (CIPN), chronic inflammatory demyelinating polyneuropathy (CIDP), and human immunodeficiency virus‐associated sensory neuropathy (HIV‐SN).

**Methods:**

A deep learning–based multiclass system was developed to analyze corneal nerve images. These images were preprocessed to train and validate the proposed model and the diagnostic utility was evaluated from the accuracy, *F1*‐score and area under the curve to derive sensitivity, specificity and precision.

**Results:**

NeuropathAI achieved excellent results: AUC—96.75%, sensitivity—83.87%, specificity—95.07%, and demonstrated excellent discrimination for CIDP, CIPN, HIV‐SN and DPN with one‐vs‐all AUC scores of 97%, 93.1%, 99.7% and 96.9%, respectively. Explainability visualization through heatmaps demonstrated that regions of decision making by the model localized to areas with nerve fiber loss, enhancing interpretability.

**Interpretation:**

NeuropathAI achieved rapid and accurate diagnosis of four of the most prevalent peripheral neuropathies globally, underscoring the potential of artificial intelligence–driven corneal image analysis for the rapid diagnosis and differentiation of peripheral neuropathies.

**Abbreviations**
AI: artificial intelligence
AUC: area under the curve
CCM: corneal confocal microscopy
CIDP: chronic inflammatory demyelinating polyneuropathy
CIPN: chemotherapy‐induced peripheral neuropathy
DPN: diabetic peripheral neuropathy
HIVSN: human immunodeficiency virus–associated sensory neuropathy
*t*‐SNE: *t*‐stochastic neighbor embedding

## 1 Introduction

Neurodegenerative diseases affect over 3.4 billion people and are associated with major morbidity and mortality [1]. Metabolic and nutritional disorders, toxins, autoimmune, infectious, and hereditary causes underlie most peripheral neuropathies and account for 10% of all consultations to a neurologist [2]. Delayed presentation and referral to a neurologist for further evaluation with quantitative sensory testing (QST), nerve conduction studies (NCS) and intraepidermal nerve fiber assessment [3, 4] lead to delayed or missed diagnosis of diabetic and other peripheral neuropathies [5].

Corneal confocal microscopy (CCM) [6] is a rapid noninvasive ophthalmic imaging technique to quantify small nerve fiber degeneration, particularly in diabetic peripheral neuropathy (DPN) [7, 8, 9, 10, 11, 12, 13, 14], but also idiopathic small fiber neuropathy [15], chemotherapy‐induced peripheral neuropathy (CIPN) [16, 17, 18], chronic inflammatory demyelinating polyneuropathy (CIDP) [19, 20], amyloid neuropathy [21] and human immunodeficiency virus–associated sensory neuropathy (HIVSN) [22]. The severity of nerve degeneration can be quantified by measuring corneal nerve fiber density, corneal nerve branch density and corneal nerve fiber length, but these measures cannot differentiate neuropathies. However, in our recent study [23] corneal nerve fractal dimension, a measure of the complexity of the remaining nerve fibers differentiated patients with DPN from those with CIPN, CIDP and HIV‐SN.

Dabbah et al. [24] initially proposed a multi‐scale dual‐model method to quantify corneal nerve fibers in CCM images to use them as feature vectors for classification using random forest and neural network classifiers. Subsequently, deep learning algorithms were applied to automatically analyze corneal nerve fibers and extract relevant features to diagnose peripheral neuropathy in participants with prediabetes and diabetes [25]. Recently [26], we deployed state‐of‐the‐art vision transformer models for binary classification of CCM images and achieved an area under the curve (AUC) of 99% distinguishing healthy controls from individuals with DPN.

In the present work, we excluded healthy controls because we and others have already established good binary discrimination between healthy individuals and patients with DPN [25, 26], CIPN [27], CIDP [19] and HIVSN [22]. Our current aim was on a more clinically relevant and challenging task: using AI to differentiate different peripheral neuropathies, where diagnostic overlap is common, and misclassification carries greater clinical consequences.

## 2 Methods

### 2.1 Study Design and Cohorts

A total of 777 CCM images were utilized from 29 patients with DPN [25], 34 patients with CIDP [19], 11 patients with CIPN [27], and 14 patients with HIVSN [22] from our previously published studies [23] applying established internationally accepted reference standard diagnostic criteria (NCI‐CTCAE): National Cancer Institute—Common Terminology Criteria for Adverse Events (CIPN); CHANT: Clinical HIV‐Associated Neuropathy Tool (HIVSN) [28]; ENFS‐PNS [29]: European Federation of Neurological Societies/Peripheral Nerve Society (CIDP) and Toronto Criteria (DPN) [30].

The dataset was carefully partitioned on a patient‐wise basis into three separate sets: 60% of the patients were used for model training, 10% were used for hyperparameter tuning (validation set), and the remaining 30% were used to evaluate the trained model's performance (testing set). This ensures that all images from a given patient appear in only one set, preventing data leakage across training, validation, and testing. This rigorous approach safeguards against the model learning and exploiting patterns specific to individual patients, thereby enhancing its generalizability and predictive accuracy on real‐world, unseen data. The CCM images, originally 384 × 384 pixels, were resized to 224 × 224 pixels for compatibility with pretrained models and normalized for faster convergence during training and to help the model generalize better to unseen data.

### 2.2 System Development

**FIGURE 1:**
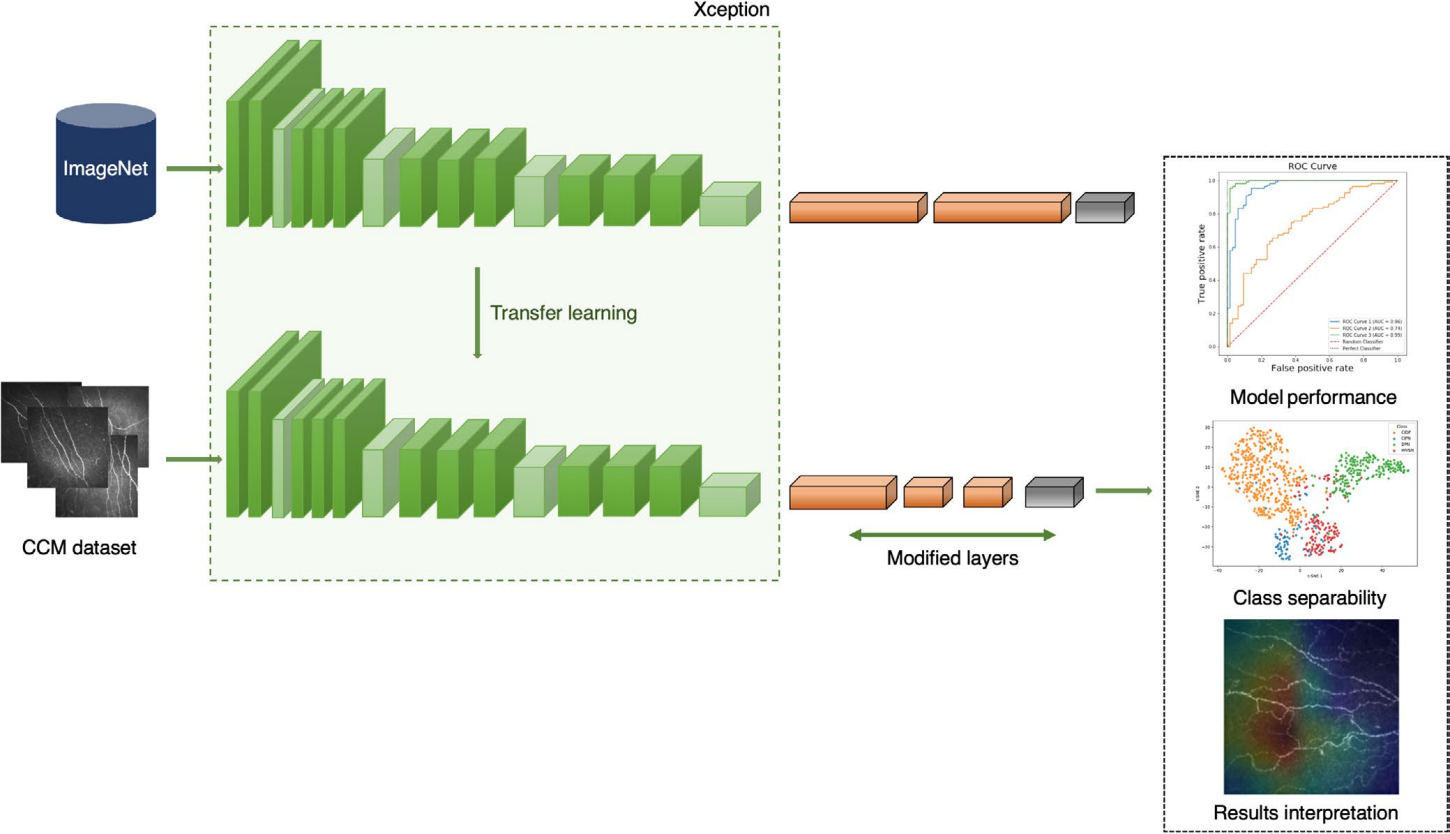
Architecture of NeuropathAI, a transfer learning–based deep learning model for neuropathological image analysis. The model leverages the Xception architecture pretrained on ImageNet (top pathway) and applies transfer learning to adapt it for corneal confocal microscopy (CCM) images (bottom pathway). The green blocks represent the Xception network's convolutional layers with varying depths, while the orange blocks indicate the modified classification layers optimized for neuropathological assessment. The right panel illustrates the model's analytical capabilities: Model performance evaluation through ROC curves, class separability and embedding structure through *t*‐SNE visualizations, and results interpretation through feature visualization heatmaps.

We designed a convolutional neural network (CNN) for image classification (NeuropathAI) (Figure 1), built using the Xception architecture [31] as its backbone. It takes an input of 224 × 224 RGB images and leverages pretrained Xception weights from ImageNet, excluding its top classification layer. The first 20 layers of Xception are frozen to retain pretrained feature extraction capabilities and reduce trainable parameters, while the remaining layers are fine‐tuned. The base model's output is flattened into a 1D vector, followed by a fully connected layer with 256 units and ReLU activation, a 128‐unit linear embedding layer, and batch normalization to stabilize training. The final output layer uses a softmax activation to produce a probability distribution for multiclass classification. Our model is well‐suited for transfer learning, combining the power of pretrained features with custom layers tailored to our specific classification task. Xception was selected as a backbone model after experimenting with other popular pretrained CNNs. Model development was conducted within the Python environment, utilizing the TensorFlow and Keras deep learning libraries. A comprehensive description of the network architecture, including hyperparameters such as filter number, kernel size, and max pooling size for each layer, is provided in the online Appendix.

### 2.3 Statistical Analysis

Data are presented as mean ± SD and the nonparametric Kruskal–Wallis test was used to assess between group differences. To assess model performance, we plotted the receiver operating characteristic curve for each class and calculated the AUC using a one‐versus‐rest approach by framing the multiclass problem as a series of binary classifications, where each class was compared against all others. Furthermore, we utilized a *t*‐Stochastic Neighbor Embedding (*t*‐SNE) [32] for visualization of model effectiveness. Model diagnoses were determined based on the probability outputs of the softmax function within the TensorFlow framework which converts logits into probabilities, and the class with the highest probability was assigned as the final peripheral neuropathy diagnosis. The evaluation was repeated across 100 random patient‐level splits of the dataset, with all images used in each split, and the average metrics including accuracy, precision, sensitivity, specificity, *F1*‐score, and AUC, across splits and classes were reported. This approach provides a more reliable representation of algorithm performance across randomly generated test datasets, rather than relying on a single split.

## 3 Results

Clinical, demographic data and diagnostic criteria are presented in Table 1.

**TABLE 1:** Clinical characteristics and diagnostic criteria employed in patients with peripheral neuropathy.

	CIPN	HIV	CIDP	DPN	*p*‐value
*n*	11	14	34	29	—
Age	62.7 ± 11.1	57.7 ± 7.8	59.2 ± 14.8	59.2 ± 14.8	0.99
Sex (M/F)	11/0	20/0	22/12	12/13	—
Diagnostic criteria	NCI‐CTCAE	CHANT	EFNS‐PNS	Toronto criteria	—

### 3.1 Model Performance

Despite evident corneal nerve loss, the visual similarity of the remaining corneal nerve fibers and lack of salient diagnostic features in each group (Supplementary Figure S1), Table S2 summarizes the resulting distributions by reporting the minimum, maximum, median, and interquartile range (IQR) of these metrics. The median sensitivity was 0.843 (IQR: 0.805–0.885), and the median specificity was 0.953 (IQR: 0.941–0.962).

**FIGURE 2:**
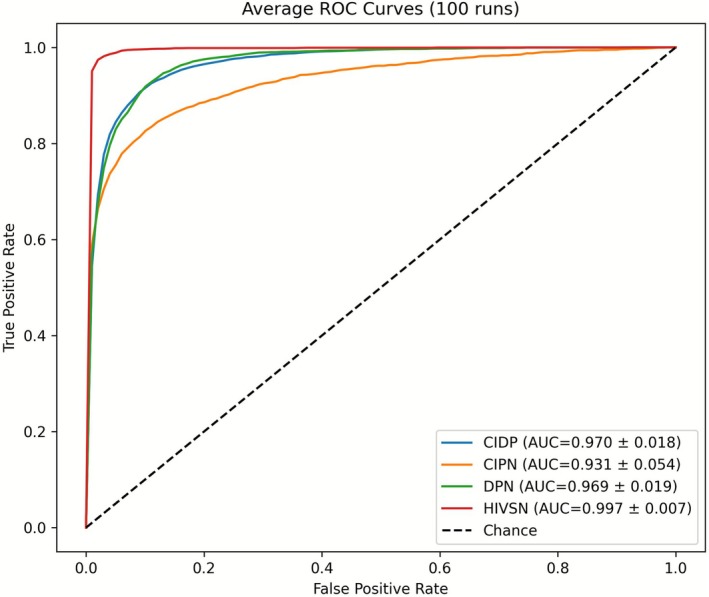
The average ROC curve for each peripheral neuropathy versus others.

The receiver operating characteristic curve (Figure 2) demonstrates exceptional performance in classifying each peripheral neuropathy with AUCs: CIPN (93.1%), HIVSN (99.7%), CIDP (97%) and DPN (96.9%). These results are further highlighted with condition‐specific metrics in Table 2, where NeuropathAI demonstrated strong performance across all neuropathy classes, with consistently high precision, sensitivity, specificity, and *F1*‐scores. For CIDP, the model demonstrated balanced and reliable performance, achieving a precision of 87.5% ± 7.0%, sensitivity of 91.7% ± 5.4%, specificity of 89.3% ± 5.0%, and an *F1*‐score of 89.3% ± 4.5%, indicating accurate identification with minimal false positives and false negatives. CIPN represented the most challenging classification task, yielding a moderate sensitivity of 65%, which suggests difficulty in capturing all positive cases. Nevertheless, the model achieved a high specificity of 97.6% ± 2.4%, reflecting a strong ability to correctly identify negative cases. The precision of 80.8% ± 14.7% and *F1*‐score of 70.0% ± 13.1% highlight this trade‐off between sensitivity and specificity. For DPN, the model achieved robust and well‐balanced results across all metrics, with a precision of 85.1% ± 9.5%, sensitivity of 82.8% ± 9.9%, specificity of 95.0% ± 3.8%, and an *F1*‐score of 83.3% ± 6.6%. The relatively small standard deviations indicate consistent model performance across validation folds. Finally, HIVSN achieved the highest overall performance, with a precision of 93.8% ± 5.7%, sensitivity of 96% ± 5.7%, specificity of 98.5% ± 1.7%, and an *F1*‐score of 94.7% ± 4.1%. The uniformly low variability across all metrics demonstrates exceptional model stability and reliability for this neuropathy subtype.

**TABLE 2:** The average classification performance using 100‐fold cross validation as assessed with precision, sensitivity, specificity, *F1*‐score and AUC for the four classes CIDP, CIPN, HIVSN, and DPN.

Class	Precision (Mean ± SD)	Sensitivity (Mean ± SD)	Specificity (Mean ± SD)	*F1* (Mean ± SD)	AUC (Mean ± SD)
CIDP	0.875 ± 0.070	0.917 ± 0.054	0.893 ± 0.050	0.893 ± 0.045	0.970 ± 0.018
CIPN	0.808 ± 0.147	0.650 ± 0.170	0.976 ± 0.024	0.700 ± 0.131	0.931 ± 0.054
DPN	0.851 ± 0.095	0.828 ± 0.099	0.950 ± 0.038	0.833 ± 0.066	0.969 ± 0.019
HIVSN	0.938 ± 0.057	0.960 ± 0.057	0.985 ± 0.017	0.947 ± 0.041	0.997 ± 0.007

### 3.2 Model Interpretability

**FIGURE 3:**
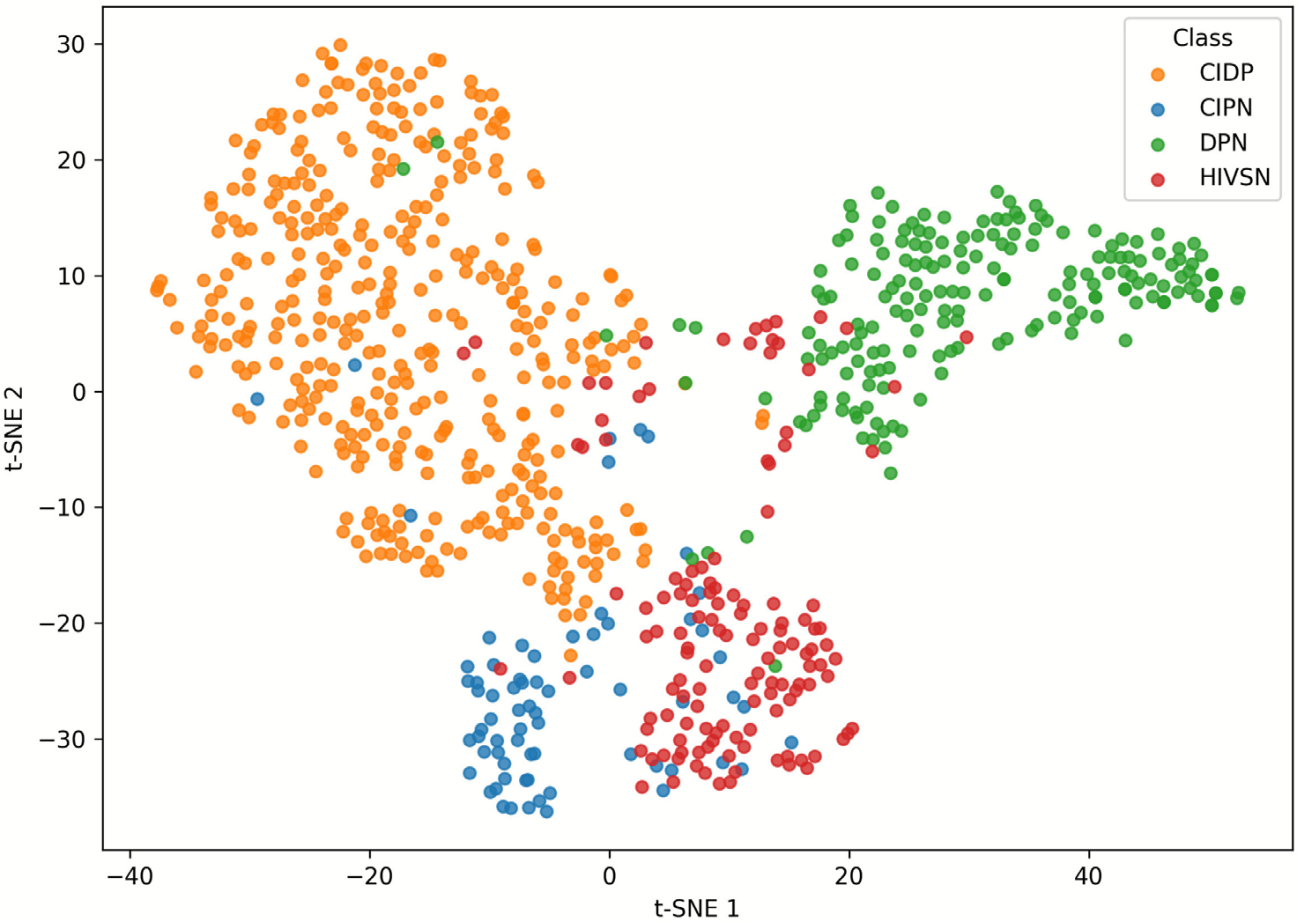
*t*‐SNE visualization of the feature embeddings learned by the model for all images across the four classes. Each point represents an image, colored according to its class (CIPN: blue, CIDP: orange, DPN: green, HIVSN: red). The plot shows reasonably well‐separated clusters, indicating that the model captures class‐discriminative features.

To objectively evaluate the ability of the model to differentiate each peripheral neuropathy, we employed the *t*‐SNE technique to generate a low‐dimensional representation of all images. The visualization reveals well‐defined clusters, indicating that the features generated by our model effectively discriminate different neuropathies (Figure 3). Furthermore, the clusters show excellent separation, indicating that each peripheral neuropathy has distinct characteristics in the reduced dimensional space our model is able to capture. Quantitative analysis of the embedding space further supports this observation: the mean intra‐class distance was 6.674, while the mean interclass distance was 10,613, yielding an inter/intra ratio of 1.59. This indicates that samples from the same class are, on average, closer to each other than to samples from other classes.

The overall silhouette score of 0.225 suggests moderate clustering quality, consistent with partial overlap observed in the *t*‐SNE plot. These results indicate that the model captures class‐discriminative features, while also reflecting some inherent similarity among certain classes. There was a small overlap between HIVSN (red) and CIPN (blue) clusters, suggesting these two conditions may share similarities in their underlying pathology, making them harder to distinguish compared to the DPN (green) and CIDP (orange) groups. This observation aligns with the clinical challenge of accurately diagnosing peripheral neuropathies, which can present with subtle and nuanced clinical features making it difficult to accurately differentiate some neuropathies.

**FIGURE 4:**
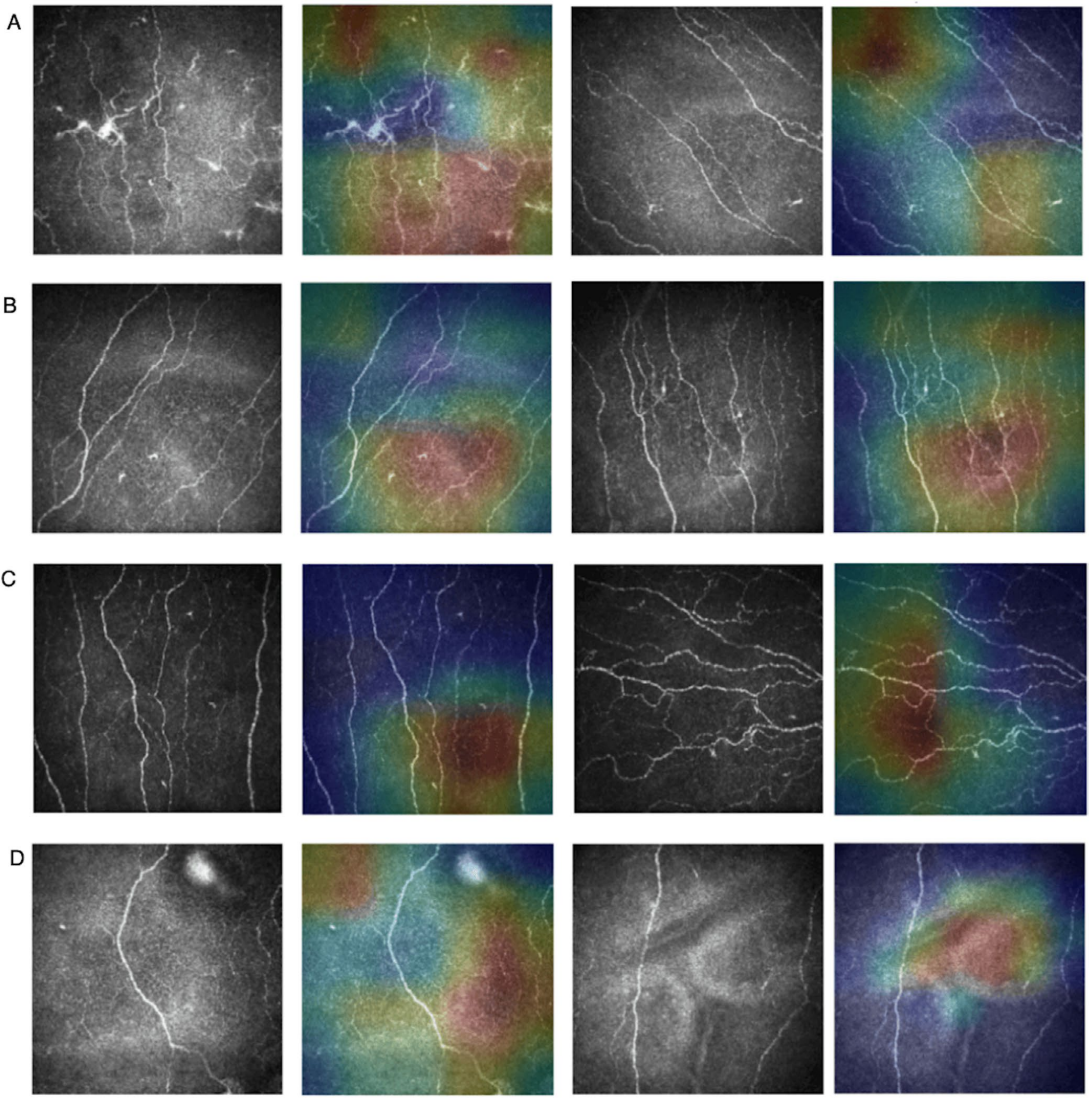
Attribution heatmaps for the multiclass problem. For each class (A)–(D) CIDP, CIPN, HIVSN, and DPN, respectively, two CCM images were selected and input to the model to generate the activation maps and gradients necessary for Grad‐CAM visualization.

Deep learning models, despite their comparable performance, often operate as black boxes, making it difficult to understand the underlying reasons for their decisions. To address this issue, we have used the Grad‐Cam technique to enhance the interpretability of detecting patients with CIPN, HIVSN, CIDP and DPN from CCM images. To identify the areas that most influenced the neural network's diagnostic predictions, we conducted an occlusion test on 72 CCM images. This test successfully highlighted the region of interest in most cases (Figure 4) and as is evident from the heatmaps the model's decision was influenced by the absence or presence of nerves. Grad‐CAM visualization primarily highlighted regions where corneal nerves were poorly detected or absent (Figure 4d). This observation aligns with the clinical challenge of diagnosing peripheral neuropathies, which can present with subtle and nuanced clinical features making it difficult to accurately differentiate some neuropathies. Furthermore, our model reached its optimal performance within only 40 epochs (Supplementary Figure S2).

## 4 Discussion

Delayed diagnosis of peripheral neuropathies can be associated with major disability. We now demonstrate a rapid, automated, explainable AI‐based ophthalmic image analysis system that highly accurately identifies and differentiates four of the commonest metabolic (DPN), toxic (CIPN), inflammatory (CIDP) and infectious (HIVSN) peripheral neuropathies in the world. This provides a noninvasive imaging paradigm and an AI‐driven method to ascertain the patho‐etiology of common peripheral neuropathies.

Diabetic neuropathy is globally underdiagnosed in up to 90% of patients with diabetes [5], even though it is associated with significant morbidity due to painful neuropathy [33], foot ulceration and amputation and a twofold increase in mortality [34]. CIPN can markedly reduce patients' quality of life and lead to chemotherapy dose reduction or discontinuation [35]. Despite established criteria to diagnose CIDP [36], there is significant clinical heterogeneity in relation to clinical course and response to treatment; atypical cases can be difficult to diagnose and indeed a significant number of patients with CIDP remain undiagnosed [37]. Human immunodeficiency virus–associated sensory neuropathy (HIVSN) is characterized by pain and paresthesia which substantially affects the quality of life [38].

Common to the diagnosis of all peripheral neuropathies, especially idiopathic small fiber neuropathy, is the identification of symptoms and neurological disability with confirmation of underlying dysfunction and damage using quantitative sensory testing (QST), nerve conduction studies (NCS) and evaluation of intraepidermal nerve fibers [39]. We have previously demonstrated reduced corneal nerve parameters in patients with DPN [40], CIPN [27], CIDP [19] and HIVSN [22] and increased immune cells in CIDP [19] and HIVSN [22]. In a recent study we showed that corneal nerve fractal dimension identified a distinct pattern in the remaining corneal nerve fibers which helped to differentiate patients with DPN and CIDP from those with CIPN or HIVSN [23].

Delayed or missed diagnosis of peripheral neuropathies can be associated with major disability. Existing evidence‐based guidelines and diagnostic algorithms are typically designed for specific neuropathies or when a particular diagnosis is already suspected clinically [39]. We now demonstrate the remarkable utility of AI in accurately identifying and differentiating four major peripheral neuropathies from CCM images alone, constituting a cost‐effective strategy. The NeuropathAI model did not require a vast database of training images and achieved excellent performance while being trained on a limited number of images, arguing for the robustness and broader application of our AI model. The incorporation of a pretrained CNN model within the model architecture was key to achieving these superior classification results and our model reached its optimal performance within only 40 epochs, highlighting its efficiency in training by rapidly converging and thus reducing the need for extensive computational resources and time. Furthermore, the occlusion sensitivity method (heatmaps) provides insights into the inner workings of the neural network and confirmed that the network identified corneal nerve fibers as key features to identify and differentiate each neuropathy.

Limitations of this study include the relatively small dataset size, which may limit the generalizability of the findings to broader populations. Further research with larger cohorts is required to confirm our findings in these and other peripheral neuropathies and rigorous real‐world evaluation is crucial, particularly in patients with rare neuropathies. A future foundation model of corneal confocal microscopy images for further fine‐tuning to specific diseases would be an advancement allowing for widespread use in both peripheral and central neurodegenerative disorders. Previous such foundation models (RETFound) have demonstrated accurate disease diagnosis and prediction in a variety of cardiovascular and neurological disorders using retinal images (color fundus and OCT [41]). Notwithstanding the practical challenge of access to corneal confocal microscopes, integration of corneal imaging alongside clinical and electrophysiological assessments may enable rapid and reliable diagnosis of peripheral neuropathies [42].

In conclusion, we have developed a novel AI model that utilizes CCM imaging to identify and differentiate with high accuracy, four of the most prevalent peripheral neuropathies globally. This aligns with our previous work demonstrating that CCM is a rapid noninvasive ophthalmic imaging tool for identifying neurodegeneration in a wide range of peripheral and central neurological diseases [43]. We believe this constitutes a rapid cost‐effective strategy for the earlier diagnosis of peripheral neuropathies by nonspecialists to enable onward referral to neurology for further diagnostic workup and timely treatment. “Corneal neuro‐oculomics” has the potential to revolutionize the diagnosis and management of patients with peripheral and central neurodegenerative diseases, especially in settings where access to experienced neurologists may be limited.

## Author Contributions

R.A.M. and A.S. conceptualised the study. M.S. generated the data and conceptualized the CIDP studies. M.F. and U.A. generated the DPN data. N.E. conceptualized the DPN study. I.N.P. and R.A.M. curated the data. C.B.R. developed the method, interpreted the results and wrote the first draft. I.N.P., A.S. and R.A.M. reviewed and verified the data and results. All authors critically reviewed the manuscript and approved the final version of the manuscript and had final responsibility for the decision to submit for publication.

## Conflicts of Interest

The authors declare no conflicts of interest.

## Supporting information


**Data S1:** acn370255‐sup‐0001‐Supinfo.docx.

## Data Availability

Access to the dataset used in this study is restricted for ethical reasons. Researchers affiliated with academic or research institutions may submit requests for access to Prof. Rayaz Malik. The source code utilized for these analyses will be made available at the time of publication throught this link https://github.com/serag‐ai/Multi‐class_CCM.git.
