# Supplementary material for: Deep Learning–Assisted Differentiation of Four Peripheral Neuropathies Using Corneal Confocal Microscopy
Source: Ann Clin Transl Neurol. 2025 Nov 22;13(4):747–54. doi: 10.1002/acn3.70255 (PMC13071145; doi:10.1002/acn3.70255)
Supplement: Supplementary file 1 — Data S1: acn370255‐sup‐0001‐Supinfo.docx. [file ACN3-13-747-s001.docx]

**Supplementary appendix**

Table of Contents

[Figure S1. 3](#_Toc210816039)

[Figure S2. 3](#_Toc210816040)

[Supplementary Methods 4](#_Toc210816041)

[Computational hardware and software 4](#_Toc210816042)

[Proposed Architechture 4](#_Toc210816043)

[Table S1. 4](#_Toc210816044)

[Table S2. 5](#_Toc210816045)

[Backbone methods 5](#_Toc210816046)

[Evaluation Metrics 5](#_Toc210816047)

[References 7](#_Toc210816048)


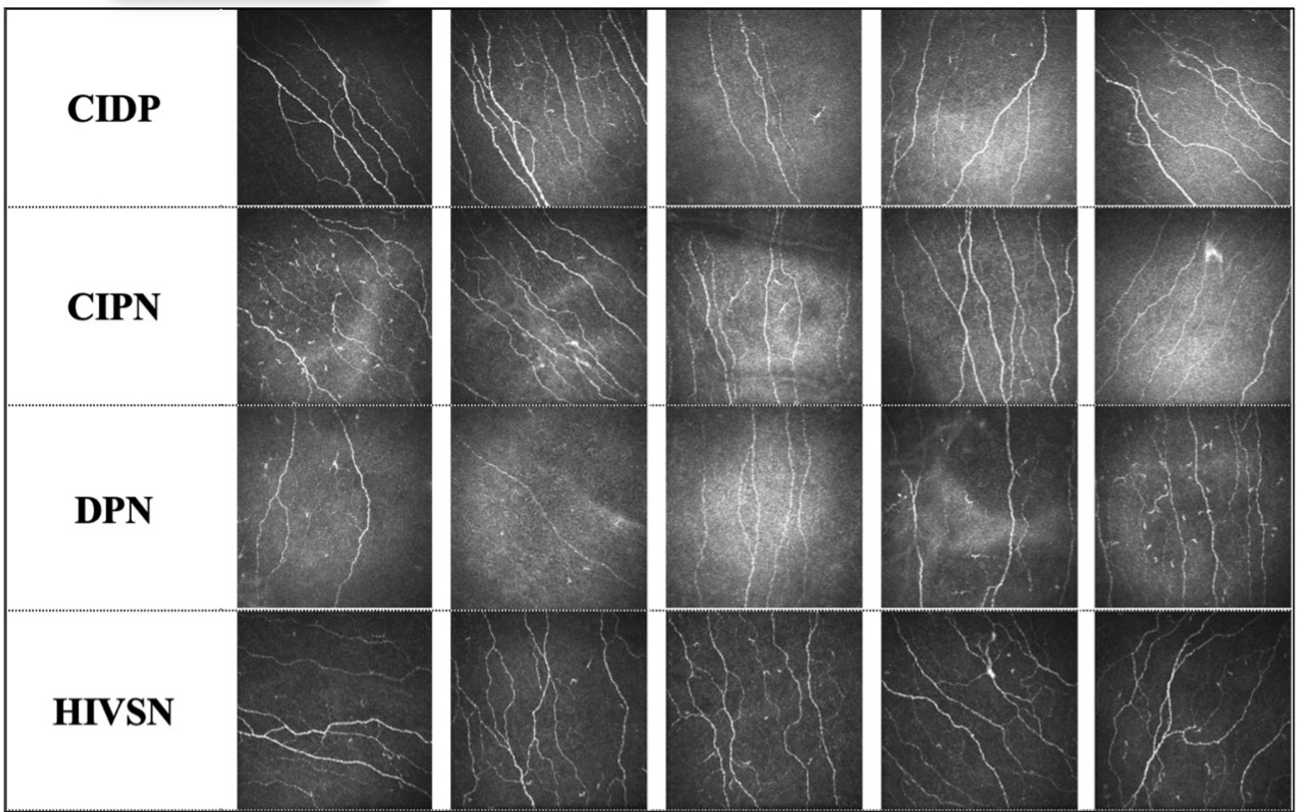


**Figure S1.** Samples of CCM images from patients with DPN, CIPN, CIDP, and HIV-SN, underscoring the considerable overlap in their characteristics, thus hindering effective differentiation and diagnosis.


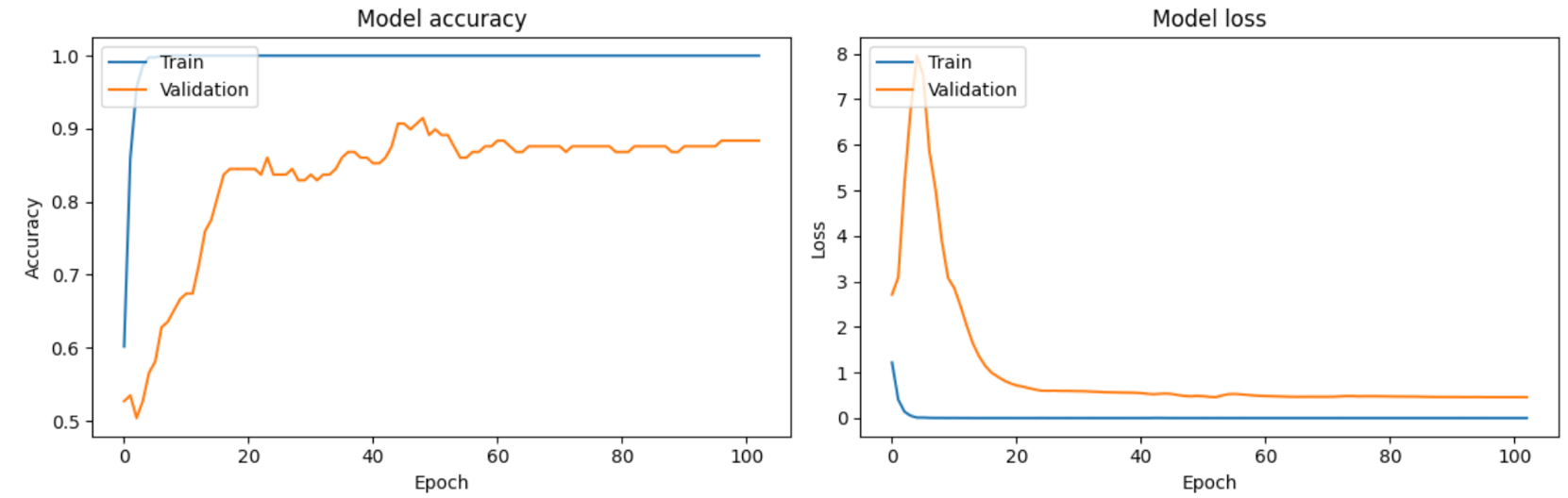


**Figure S2.**  Training and validation accuracy and loss curves over number of epochs

# **Supplementary Methods**

## **Computational hardware and software**

We stored all images in a bitmap (bmp) or (JPEG) format in a (High Performance Computing) cluster with total a size around 129MB. The training of the models was performed on a GPU P100. The whole code was written in Python (version 3.12.7), with the following external package: matplotlib (3.9.2), numpy (2.3.3), pandas (2.3.3), scikit-learn (1.7.2), scipy (1.16.2), tqdm (4.66.5).

## **Proposed Architechture**

NeuropathAI leverages the power of transfer learning for a four-class image classification task, using the Xception architecture as its foundation. It begins with 224x224 RGB images as input. A pre-trained Xception model, pre-loaded with weights from ImageNet, serves as the base for feature extraction. To balance leveraging pre-trained knowledge with task-specific learning, the initial 20 layers of the Xception model are frozen, preserving the robust feature extraction capabilities learned from ImageNet and significantly reducing the number of parameters requiring training. The remaining, unfrozen layers of Xception are then fine-tuned on the target dataset, allowing the model to adapt its learned features to the nuances of the specific classification task.

The output from the Xception base is flattened, converting the multi-dimensional feature maps into a one-dimensional vector. This flattened vector is then fed into a fully connected (dense) layer of 256 units, activated by ReLU for non-linearity. Following this, a linear embedding layer with 128 units further reduces the dimensionality of the feature representation. A batch normalization layer is incorporated after the embedding layer to stabilize and accelerate the training process. Finally, a fully connected output layer with four units (corresponding to the four classes) and a softmax activation function generates a probability distribution over the four classes, providing the model's classification predictions. This architecture effectively combines the power of a pre-trained Xception model with task-specific fine-tuning and additional classification layers to perform multi-class image classification.

The model is compiled with the Adam optimizer, categorical cross-entropy loss, and metrics for accuracy and AUC. Callbacks are used to stop training early if no improvement is observed in validation loss for 50 epochs.

The learning rate is reduced if no improvement is observed in validation loss for 15 epochs.

The hyperparameters values are given in Table S1.

**Table S1.** Proposed Network Hyperparameters.

| **Hyperparameter** | **Value** |
| --- | --- |
| Loss Function | Categorical Cross-entropy |
| Optimizer | Adam |
| Batch size | 64 |
| Max number of epochs | 500 |
| Learning rate | 0.001 |

**Table S2.** Performance across 100 runs reporting the minimum, maximum, median, and interquartile range (IQR) of the AUC, sensitivity, and specificity.

| Metric | Min | Max | Median | Q1 (25%) | Q3 (75%) | Mean | Std | IQR |
| --- | --- | --- | --- | --- | --- | --- | --- | --- |
| **AUC** | 0.8572 | 0.9932 | 0.9706 | 0.9586 | 0.9826 | 0.9675 | 0.0202 | 0.0241 |
| **Sensitivity** | 0.6052 | 0.9435 | 0.8430 | 0.8052 | 0.8853 | 0.8387 | 0.0587 | 0.0801 |
| **Specificity** | 0.8753 | 0.9833 | 0.9530 | 0.9409 | 0.9616 | 0.9507 | 0.0171 | 0.0208 |

## **Backbone methods**

In the following, we give an overview of the implementation details of each of the backbone models tested. All these models are pre-trained on ImageNet dataset[1]

**VGG16**

VGG16[2] is a CNN architecture that was introduced by the Visual Geometry Group (VGG) at the University of Oxford in 2014. It gained popularity due to its simplicity and strong performance on image classification tasks, particularly on the ImageNet dataset, which contains over 14 million images across 1,000 classes. The deep architecture and small filters enable VGG16 to learn hierarchical features, from edges and textures in early layers to complex patterns and objects in deeper layers.

**InceptionV3**

InceptionV3[3] is a CNN architecture that has been shown to be highly effective for image classification tasks. It is a deep learning model that is part of the Inception family of models, which are known for their ability to efficiently analyze complex visual data.

**ResNet50**

ResNet50 belongs to the family of ResNet [4]. It is a 50-layer deep residual network that utilizes skip connections to facilitate the training of deeper architectures. By allowing information to bypass multiple layers, ResNet50 can effectively learn complex patterns and achieve higher classification accuracy.

**DenseNet121**

DenseNet121[5] is a powerful and efficient CNN architecture that leverages dense connections to improve feature reuse, reduce parameters, and enhance gradient flow. It is widely used for image classification and transfer learning tasks, offering strong performance with relatively low computational cost. Its innovative design makes it a popular choice in both research and practical applications.

**Xception**

Xception [6] short for Extreme Inception, is a deep learning model that extends the Inception architecture. While Inception models employ inception modules, Xception utilizes depthwise separable convolutions. This approach significantly reduces computational cost while maintaining high performance. The model consists of 36 layers, enabling it to capture intricate visual patterns.

# **Evaluation Metrics**

The majority of metrics employed utilise four evaluation metrics:

- True Positive (TP): A positive instance that was correctly predicted as positive.
- True Negative (TN): A negative instance that was correctly predicted as negative.
- False Positive (FP): A negative instance that was incorrectly predicted as positive.
- False Negative (FN): A positive instance that was incorrectly predicted as negative.

**Confusion matrix**

The confusion matrix is a clear and informative metric for evaluating the accuracy and correctness of a DL algorithm in classification tasks. It summarizes the number of samples for each combination of true and predicted classes. Typically, columns represent true labels, while rows represent predicted labels.

**Sensitivity**

Sensitivity is the proportion of actual positives that are correctly identified as positives by the model.

$$Sensitivity= \frac{TP}{TP+FN}$$

**Specificity**

Specificity is the proportion of actual negatives that are correctly identified as negatives by the model.

$$Specificity= \frac{TN}{TN+FP}$$

**F1-score**

The F1-score, also known as F-measure, is a metric that evaluates the algorithm performance where sensitivity measures the proportion of correctly predicted positive instances among all actual positive instances. A high F1-score indicates that the classifier effectively minimizes both false positives and false negatives.

$$F1= \frac{2TP}{2TP+FP+FN}$$

**Precision**

Precision measures the proportion of correctly predicted positive instances out of all instances predicted as positive.

$$Precision= \frac{TP}{TP+FP}$$

**Accuracy**

Accuracy measures the proportion of correctly classified instances to the total number of observations. It is a commonly used metric for evaluating classification models. However, accuracy can be misleading in imbalanced datasets where one class significantly outnumbers the other. Therefore, it’s often used in conjunction with other metrics to provide a more comprehensive assessment of model performance.

$$Accuracy= \frac{TP+TN}{TP+FN+TN+FP}$$

**Area Under Curve**

The area under the ROC (Receiver Operating Characteristics) curve, called AUC, has been a standard metric in medical diagnosis since the 1970s. A ROC graph plots the TP rate on the Y-axis and FP rate on the X-axis. Recently, it has gained popularity as a comprehensive measure for evaluating the predictive performance of DL models. In general, AUC is favored over accuracy.

# **References**

1. Deng J, Dong W, Socher R, Li LJ, Li K, Fei-Fei L. Imagenet: A large-scale hierarchical image database. In2009 IEEE conference on computer vision and pattern recognition **2009** Jun 20 (pp. 248-255). Ieee.
2. Simonyan K. Very deep convolutional networks for large-scale image recognition. arXiv preprint arXiv:1409.1556. **2014**.
3. Szegedy C, Vanhoucke V, Ioffe S, Shlens J, Wojna Z. Rethinking the inception architecture for computer vision. InProceedings of the IEEE conference on computer vision and pattern recognition **2016** (pp. 2818-2826).
4. Koonce, B.; Koonce, B. ResNet 50. *Convolutional neural networks with swift for tensorflow: image recognition and dataset categorization* **2021**, pp. 63–72.
5. Huang G, Liu Z, Van Der Maaten L, Weinberger KQ. Densely connected convolutional networks. InProceedings of the IEEE conference on computer vision and pattern recognition **2017** (pp. 4700-4708).
6. Chollet, F. Xception: Deep learning with depthwise separable convolutions. In Proceedings of the Proceedings of the IEEE conference on computer vision and pattern recognition, **2017**, pp. 1251–1258.
